# Comparing the reasons for suicide from attempt survivors and their families in Ghana

**DOI:** 10.1186/s12889-019-6743-z

**Published:** 2019-04-16

**Authors:** Winifred Asare-Doku, Joseph Osafo, Charity S. Akotia

**Affiliations:** 1 School of Medicine and Public Health, University of Newcastle, Callaghan, NSW 2308 Australia; 2 Department of Psychology, University of Ghana, Legon, Ghana

**Keywords:** Suicide, Attempt survivors, Families, Ghana

## Abstract

**Background:**

Family members are key in detecting early suicide warning signs. This study compared the reasons for suicidality as reported by attempt survivors with their family folks in Ghana.

**Methods:**

A qualitative design was used to select a sample size of 20 (10 family members and 10 suicide attempt survivors). Thematic analysis was used to analyse the data.

**Results:**

Analysis of the data showed two major themes: 1) Similar Reasons (where both families and attempt survivors consistently reported similar reasons for suicidality and 2) Different Reasons (where there were sharp divergences).

**Conclusions:**

Findings from this study help to understand the readiness of attempt survivor family members to intervene early during suicidal crisis.

**Electronic supplementary material:**

The online version of this article (10.1186/s12889-019-6743-z) contains supplementary material, which is available to authorized users.

## Background

The interpersonal theory of suicide explains that there needs to be the desire and ability to kill oneself for suicidal behaviour to occur [1]. Joiner [1] further explains that social disconnection or alienation and perception of being a burden to others are reasons for why suicide occurs. According to the theory, social disconnection and burdensomeness is influenced by the mental state of individuals, and this leads to suicidal ideation. Various studies have examined the reasons for suicidality as reported by attempt survivors and significant persons around them such as health workers and families [2, 3]. Some studies have examined perspectives on why suicide occurred and in one of such study, a majority of the patients reported impulsivity and loss of a sense of control as reasons for their action, but some doctors and nurses viewed the act as impulsive [4]. Also, patients described anxiety and panic before the attempt, which was least mentioned by the doctors; the doctors and nurses reported despair and hopelessness before the attempt [4]. In another study, general practitioners and psychiatrists favoured medical explanations, but the general population and suicide attempt survivors favoured psychosocial explanations for attempting suicide [5]. Thus, suicide attempt survivors understand their attempt differently than mental health professionals who treat them.

Suicidal behaviour disrupts the family system. There are however limited studies on families’ experiences or reactions after a suicide attempt [6, 7]. Examining the perspective of family members is essential to better understand the treatment and prevention of suicidality in at-risk individuals [8]. This is important because the family system also plays a role in the development of suicidal behavior [9].

Studies in Ghana have reported reasons for suicide including shame [10], faith crisis [11], stigma, social exclusion, perceived infidelity, social taunting [12], betrayal, financial problems, neglect, and existential crisis [13, 14]. Until now, studies examining reasons for suicide have focused only on attempt survivors or mental health professionals, without any narratives from the family. Although attempt survivors’ view on the reason/s for suicidal behaviour might not be contested, it will, however, be relevant to find out what their families also think about this for purposes of gauging the latter’s readiness for support and early intervention. Further, the views of the families are relevant in understanding suicide [15] because they highlight the importance of early identification of warning signs in prevention and intervention programs [16]. There are reliable indicators that a suicide attempt leaves suicide persons and families at loggerheads, with strong evidence of dyadic tensions, which may lead to an escalation of distress among suicidal persons [6]. This presents opportunities to compare the views of attempt survivors directly with their families on the reasons for the act. The purpose of the study is to explore the reasons for suicidal behaviour from the perspective of attempt survivors and compare them with the views of their immediate family. The importance is to observe potential disparities and non-disparities in the narratives, which may reflect obliviousness on the part of the family in understanding what “actual” reason for the suicidal act is. The importance of doing this study is to show where the perspectives of people who attempted suicide and their families are convergent and where they are not. Knowing that would help clinicians to help both patients and families on how to promote effective communication during a suicidal crisis. This will also highlight the need for intervention on how to detect early signs for suicide and provide immediate help.

## Method

### Participants

We recruited participants in Accra, the capital of Ghana, at the Psychiatry Department of Korle-Bu Teaching Hospital (KBTH). The KBTH is a leading national referral centre in Ghana. We retrieved a contact list of 40 suicide attempt survivors from the records of the psychiatric unit. All phone numbers on the list were called, and for those we had a response from, they were informed about the purpose of the research. Out of the 40-contact list, 10 consented, four people declined to participate, 18 of the contact numbers did not go through, six of the contact did not respond, and two consented and later declined. Ten participants consented to participate and were recruited for the study. At the time of the interview, eight of the participants were discharged from the hospital, one was outpatient, and the other was inpatient. One patient was diagnosed with bipolar. Criteria for inclusion were over age 18 years, survived a suicide attempt six months or more prior to the interview. Survivors were aged 18–34, and half were students. Two (one female and one male) of the attempters reported more than one attempt.

Additionally, we contacted close relatives such as parents and siblings referred to us by the participants. The participants were informed that an interview would be conducted with a relative who was available. A relative consisted of any member of the household or by blood to the suicide attempter who has lived with the suicidal person for at least two years. We interviewed relatives who consented. This process resulted in 20 participants for this study; 10 attempt survivors and 10 relatives.

### Procedure

The main instrument for data collection during the interview was a semi-structured interview guide (see ‘Additional file 1’ for interview guide). For instance, one question for the attempter was, *Why did you engage in the suicidal behaviour*? Also one question for the family member was, *Why do you think your relative attempted suicide?* In strongly proscriptive contexts such as Ghana, these are questions that help us understand the cultural dynamics involved in the reasons for the act and potential barriers that may impede the readiness of families to provide early help.

The interview guide was either in Twi (one of the most widely spoken Ghanaian languages) or in English as needed. Some interviews were in participants’ homes and some were at the psychiatry department, which is devoid of distractions and where people may not eavesdrop. We interviewed attempt survivors first and then interviewed at least one family member of the attempt survivor who was available and willing to participate. Their interviews were conducted in a place where there was no distraction. When relatives were unavailable at the same time, we scheduled them within at least a week. Averagely, the interviews lasted between 40 min to an hour.

We obtained the permission of the institutional review board- Ethics Committee for Humanities, at the University of Ghana to conduct this research. Participants signed an informed consent form that told them that they could withdraw from the study at any time. Participants’ consent covered participation and dissemination of the findings of the study. We kept all data confidential. Two of the participants experienced psychological and physical distress because they had to recall past events. We truncated these interviews, continued them later, and provided psychological support for them. As part of debriefing, we re-contacted participants eight weeks after the interview to ensure that they were doing well. Many reported that the interviews provided them with the first avenues to share their untold stories for their suicidality with a third party, someone they perceived non-condemning.

Thematic analysis was used in developing themes [17]. This was done by familiarising with the data. We generated initial codes after this and observed patterns in the data through the codes. This level focused on the broader development of themes and sorting codes into themes. Further, we refined the themes developed and explored the connections between them. [17]. The first author conducted the interviews and generated the initial codes for further discussions with the second and third author. The second and third author challenged the initial codes and provided inductive analytical rigour through extensive discussion of codes until all authors reached consensus.

## Results

We obtained two major themes: *similar* and *different reasons* (see Table 1 for summary of themes) *.* For the purposes of confidentiality and anonymity, we state only participant’s gender, age, and whether the participant was an attempt survivor, (SA) or family (F) at the end of a quote. Other details of participants are reported in the table below (see Table 2).

**Table 1:** Summary of themes

Attempt survivors’ reasons for suicide	Relatives’ reasons for attempt survivor’s suicide
Similar reasons	
My stepmum used to beat me and insult me. (SA, Female, 18 years)	Her stepmum was worrying her and wanted me to sack her from the house. (F, Male, 62 years).
Then all of a sudden he packs his things and goes to stay with another woman (SA, Female, 28)	The man she wanted to be with did not work out too (F, Male, 50)
I knew that so long as the money was missing, my life had ended (SA, Male, 27 years).	He said thieves had taken his money and he wanted to kill himself*.* (F, Male, 45 years).
At home they trouble you and treat you as if you are nothing to them (SA, Male, 21 years).	My elder sister also doesn’t like him, she has been talking to him anyhow and insulting him (F, Male, 25 years).
I was having arguments with him (her boyfriend) on this fateful day.. (SA, Female, 34 years).	I knew she was having problems with her boyfriend and his family.. (Female, 30 years).
Different reasons	
I was having a disagreement with my girlfriend over a trivial issue (SA, Male, 24 years).	When my husband and I have quarrels, I realized it affects him.. (Female, 56 years).
My husband and I were not on good terms then (SA, Female, 34 years).	I spoke to some people a long time ago about it and told them I suspected my wife had a mental problem (F, Male, 44 years).
My mum and I had a deal and she said she doesn’t have any money. I had put my hopes high (SA, Male, 20 years).	The other was that feeling that all was lost because of his school grades (F, Female, 30 years).
I will call it depression and isolation (SA, Female, 24 years).	I don’t have any idea and to my best of knowledge there was nothing wrong with her (F, Female, 40 years).
I think it was spiritual or something because I wasn’t having any problem with anyone (SA, Female, 21 years).	I knew that she didn’t have much friends, she was always alone. Am sure that caused it. She didn’t tell the truth. I don’t think it is spiritual (F, Female, 31 years).

**Table 2:** Demographic characteristics of suicide attempters and their families

Attempters	N (%)	Family of attempters	N (%)
Males	4 (40)	Male	6 (60)
Females	6 (60)	Females	4 (40
18–25	6 (60)	25–45	5 (50)
26–34	4 (40)	46–62	5 (50)
Student	5 (50)	Civil servant	5 (50)
Courier service	1 (10)	Artisans	2 (20)
Artisans	3 (20)	Security officer	2 (20)
		Courier service	1(10)
Poisonous substances	8 (80)		
Rope	1 (10)		
Slit wrist	1 (10)		

### Similar reasons

There were parallels between attempters and family members in the reported reasons for the suicide attempt. In five cases, agreed-upon reasons/subthemes were domestic abuse, emotional pain with sense of loss, sense of worthlessness and romantic tensions.

#### Domestic abuse

The stepmother abused the suicide attempt survivor incessantly and was likely to have triggered the thought to attempt suicide as a means of escape. For example, one survivor reported abuse from her stepmother:

*My stepmum used to beat me and insult me. When it happened, I run away to my grandparent’s house, so that my parents can have peace at home. My parents had travelled at the time I left. When they returned…and were asking of me, neighbors around told them they had not seen me. He called my phone but I refused to pick the call... However, I told my grandparents that am not going back to my dad because of the way the woman was treating me in the house. My granddad accompanied me back home and they talked about everything but still she did not change. The woman was always saying that the man am living with is not my dad so I shouldn’t say he is my dad...she kept insulting and saying bad things about my real mum so one day I took a disinfectant from another woman’s kitchen and drank it* (SA, Female, 18 years).

This survivor indicated that attempts to get the stepmother to relate well failed; further, neighbours’ intervention did not help. She became sad and wanted to die.

This attempter’s father validated the poor relationship with her step-mum as narrated below:

*I honestly do not have any idea why she did that because she is my daughter and I provide all her needs. So I don’t know why she attempted. I had travelled and was told when I returned that she run away. Her stepmum was worrying her and the step mum wanted me to sack her from the house but I objected. The stepmum rather left the house. We are divorced now* (F, Male, 62 years).

#### Emotional pain with sense of loss

In this theme, suicide attempters experienced a sense of loss such as a valuable relationship or money, which caused them much distress. One attempt survivor said:

*My uncle was trying to connect me with a white man so I marry him and leave Ghana with him. However because my husband (He was not my husband then) had helped me a lot and gives me money and feed’s me, I didn’t want to disappoint him by going to take another man. He also threatened me that I shouldn’t leave him because of all that he had done for me...Then all of a sudden he packs his things and goes to stay with another woman whiles we are living together. So, it was like he didn’t allow me to marry who my uncle was planning for me and on the other hand, he is chasing another woman so it was like I had lost both opportunities. It was during that time that I felt the pain and anguish, and we had disagreements most times...* (SA, Female, 28)

A family member agreed, saying: *I was not angry with her although I was hoping she will marry someone I was planning for her. The man she wanted to be with did not work out too. But I can’t really say what might have happened. I am not living with her* (F, Male, 50).

A different kind of loss was money. One attempter said:

*Money got missing at home because thieves came to steal the money at home. The money belonged to me and someone else. I searched and searched for the money but couldn’t find it... The money was 5000 cedis ($1250*). *..I knew that so long as the money was missing, my life had ended. I didn’t tell the person that this was what had happened* (SA, Male, 27 years).

A male relative agreed:

*I was not around when it happened so I don’t know what really could have caused it, I was in Kumasi (capital town of Ashanti Region in Ghana). I was sad when I heard it. He said thieves had taken his money and he wanted to kill himself. I pray that something like this would not happen again* (F, Male, 45 years).

#### Sense of worthlessness

An attempt survivor did not feel important or included in his family, which he explained, was due to his inability to contribute at home. One attempter said:

*...At home they trouble you and treat you as if you are nothing to them. They [family members he lives with] make you feel you are hopeless. They don’t respect you and anybody speaks anyhow to you. They took me for granted because they feed me at home, so they speak anyhow to me and want to tell me what to do. Because I don’t contribute anything at home, my friends can’t even visit me at home unless I visit them. My sister was also worrying me and insulting me all the time and speaks anyhow to me. The first thing that came to mind before I drank the disinfectant was that, I mean nothing to these people and they don’t appreciate anything I do for them* (SA, Male, 21 years).

A male sibling added that the attempt survivor is hypersensitive but confirmed that the single straw that led to the attempt was his distress about the way his sister treated him:

*The reason was that he was not having a better job and also he was at home always. My elder sister also doesn’t like him, she has been talking to him anyhow and insulting him. As for my brother I know him very well, he feels pain in everything. She has been troubling him always. So I think it’s because of my sister that’s why he attempted* (F, Male, 25 years).

#### Romantic tensions

Some attempters reported dyadic tension as the reason for their suicide attempt. For example:

*I was in a relationship with a guy, and the rent of house I was living in with my children was due... we didn’t get any other place to rent so the guy I was dating took us to his house. Before I moved there, I was on good terms with the guy’s family. But just the day we moved in, the family fought with the guy and even when I greeted them, they didn’t respond well...The guy’s behavior later began to change. Sometimes he will frown at me and not talk to me for no reason...I was having arguments with him on this fateful day and that day too my daughter went to do something she shouldn’t have done so I was really angry and the insults were too much.* (SA, Female, 34 years).

Her sister said:

*I don’t know why it happened but I knew she was having problems with her boyfriend and his family. Maybe that could have caused it. I was sad because she is the only sister I have and she wants to kill herself.* (Female, 30 years).

### Different reasons

There were also dissimilar reasons between the narratives of attempters and family members with regards to the suicide attempt. In five cases, survivors’ narratives differed from relatives; difficult romantic relationships versus difficult family relationships, difficult family relationships versus mental illness, family tensions versus underachievement, mental illness versus nothingness and diabolism versus difficult relationships.

#### Difficult romantic relationships versus difficult family relationships

The suicide attempter and family disagreed on what led to suicide attempt. Whereas the suicide attempter reasoned that difficult relationship with his girlfriend led to the attempt, the family member intimated that it was as a result of issues at home.. For example, a student (an attempt survivor) implicated difficulties in his romantic relationship with his girlfriend as a reason for suicide attempt:

*I was having a disagreement with my girlfriend over a trivial issue, so I didn’t understand why the issue had become a problem. I just don’t like to have problems and is like if problems come I get all agitated, and ask myself why this is happening. So I really didn’t know what was happening… for a moment I didn’t know what I was doing. Okay! That was the trigger. Already I have a whole lot of back load things that has happened* (SA, Male, 24 years).

His mother disagreed, saying:

*...for my son I don’t know of any reason that could have caused it. When my husband and I have quarrels, I realized that it affects him. So maybe that could have caused it but am not sure* (F, Female, 56 years).

#### Difficult family relationships versus mental illness

In another case, the survivor described difficult relationships with husband, a cousin, and a brother as reasons for her suicidality:

*My husband and I were not on good terms then. My cousin came to stay with us to school and when she came, she was very disobedient. No one could control her...I was against some of the things she did but my husband supported her so she took me for granted...my cousin misbehaved and I called my brother to inform him that I want the girl (cousin) to leave our house. He advised against it and promised to talk to her to change. That evening I called my brother again and told him I didn’t see any change in her since that morning. Just then, my husband took the phone from me and told my brother that it’s not that the girl is not good but I am rather not the good person. Honestly, that statement worried me and before all this, I was not on good terms with my husband as I have already told you. So it made my brother believe what my husband told him. When I took back the phone my brother told me that if I don’t keep quiet and let the girl stay with us and finish her school, then he ceases to be my brother and I should not call him again. So because of what my brother said and when I look at the behavior of the girl who is going to live with us for three years before she completes school, I can’t stand it. And since my brother said if I do not allow her to stay, he ceases to be my brother I had to make a decision...i wanted to die* (SA, Female, 34 years).

Her husband contradicted her, reporting mental illness instead:

*I was really confused and suspected it was a mental problem when it happened. I didn’t think she could go to that extreme to kill herself. I spoke to some people a long time ago about it and told them I suspected my wife had a mental problem. They didn’t believe me. I once suggested we go to see a psychiatrist and she got angry with me. She said am thinking she is mad. So when she attempted to kill herself, I told the people that my suspicions were right. Actually, I later noticed why she did it after doing some research and visiting some experts. She was mentally and emotionally not well. She was not able to regulate such emotions. So the emotions push her to do that. That time she was hopeless and thought she was rejected so it’s better she will not live*. (F, Male, 44 years).

#### Family tensions versus underachievement

In another case, the attempter presented multiple issues that led to his suicidality:

*I had come home on that Wednesday evening…my mum and I had a deal that every week she would give me like 50 cedis ($15) for the week. Therefore, when I came home I was asking her about the money...and she said she doesn’t have any money... When she said that I was angry because I had put my hopes high...I was really upset when she said that. I told her that if she doesn’t have the money for me am going to burn her cloth as repayment... So I took a match and lighted it with a newspaper and took the cloth and threw it away. It was a new cloth... So that was when my uncle, the one am not close to came around...another uncle asked me whether I was the one burning my mum’s cloth and I said yeah and he slapped me and pushed me. Later he hit me again... so I was like okay, if I end my life I won’t be blamed for everything and no one can complain that I have done this or that. Everyone will have their peace of mind. So I just went to the kitchen and I saw a knife and took it and was slitting my wrist...My uncle had said some time ago that even if I was dying and he was there alone, he will never help me. So that was also another reason why I wanted to end it all...It’s like every time something comes up, [I] am the reason and everyone keeps talking about it. All these were part of the reasons why I wanted to kill myself. I also felt in a way that my sister had neglected me because we were not talking as much as we used to talk. She is married now and with her husband so right now her focus will be her husband* (SA, Male, 20 years).

His sister disagreed, saying that one reason for his suicide attempt was a search for attention. She thought another reason was school underachievement:

*I strongly believe that one was the attention he was not getting. I believe he did not really want to die. The other was that feeling that all was lost because of his school grades and his friends were moving on and he was home* (F, Female, 30 years)*.*

#### Mental illness versus nothingness

One survivor implicated depression and isolation as reasons for her suicidal behaviour:

*I will call it depression and isolation. That’s the most general term I can give you. I had a series of things bothering me. Am all alone taking care of me and fending for myself. Nobody supports me no friend. The love from outsiders and activities just went off automatically. I didn’t have the zeal to move on again. I will scroll through my phone repeatedly and there was no one to talk with. Before it happened, I was complaining of insomnia. I was not sleeping and I come to school with heavy eyes. I was not sleeping for a very long time. It just happened... I wanted to die* (SA, Female, 24 years).

Her sister disagreed:

*I don’t really know what caused the attempt. She is in school and we were told what happened. She is provided with her needs. I don’t have any idea and to my best of knowledge there was nothing wrong with her* (F, Female, 40 years).

#### Diabolism versus difficult relationships

In another case, a woman attributed her attempt to spiritual forces beyond her control:

*I think it was spiritual or something because I wasn’t having any problem with anyone. Like my dad didn’t do anything to me and it was not like I was pregnant or what people normally do or broken hearted or something. Before that day, I was having series of nightmares and I saw my mother calling me to come to her although she was dead. But what really happened that day before I went to take in the medicine, I can’t tell...When I woke up after I was in the hospita*l... (SA, Female, 21 years).

Her aunt, however, thought she was frustrated and lacked good social relationships:

*I was not around when it happened and when I came and heard that this is what she had done, I tried asking her. She was like she will tell me what happened later, but I asked her other times and she didn’t tell me. Later I found out and she was telling the family that she did it because her mum who is dead was calling her to come. She saw her mum’s ghost or whatever calling her to come. But me when I heard that, I was like it’s not true. It could be that she was frustrated over something that caused it. Because I knew that she didn’t have much friends, she was always alone. Am sure that caused it. She didn’t tell the truth. I don’t think it is spiritual*. (F, Female, 31 years).

## Discussion

The age range of the attempt survivors (18–34) was generally closer to the population of young adults 15–29 with the highest risks for suicidal behaviour and death as reported by the WHO [18]. Within such group, suicide accounts for 8.5% of all deaths and is ranked a second major cause of death following motor-traffic accidents [18]. Another critical demographic information is that half of the participants were students, and some reports in Ghana show that suicidality is common among school going children [3]. Ingestion of poisonous substances was the most standard method used, which diverges from other studies in the country reporting hanging [3, 18]. However, it is consistent with WHO’s report of the conventional methods for suicide including poisonous substances such as pesticides [18]. The WHO reported that previous suicide attempt is the single most important risk for suicide in the general population [18]. The risk for suicide is elevated 40 to over 100 times among attempt survivors compared with that in the general population [18, 19]. Thus, the attempt survivors for this study are high-risk individuals and need support from those around them including their families.

In comparing reasons for suicidality, a simple criterion used to assess the similarity of the attempt survivors’ narratives with that of their relatives was that a relative should confirm at least one precipitant in the narrative of the former. In five cases, the views of the families almost confirmed the reasons for the suicidal behaviour as narrated by the attempt survivor. In this study, suicide attempters implicated psychosocial stressors more than psychiatric conditions as the primary reasons for their attempt. Although in two cases, family relations suspected a psychiatric condition, the attempt survivor implicated difficult interpersonal issues accounting for her suicidality.

Findings from this study are consistent with other studies in Ghana on reasons for suicide include financial problems, existential crisis, and relationship crisis [13, 14]. Interpersonal and family relationships affect health in ways that relate to the risk for suicide [9]. The interpersonal theory of suicide can be used to explain some of the reasons intimated by suicide attempters. Some of the reasons cited in the study included hopelessness and emotional pain, and this can lead to loneliness leading to suicidal behaviour. This can be seen in the study where some participants indicated that it was better for them to die which serves as a form of escape.

Further, consistent with other studies, psychosocial strains contribute to suicide attempts in Ghana as also reported in certain Low and Middle-Income Countries (LMIC) settings [12, 13, 20–24]. In addition to this, participants may be avoiding a stigmatising label of mental illness by emphasising the importance of psychosocial factors leading to a suicide attempt. This may be an advantageous strategy for avoiding the stigma associated with mental illness.

Although several factors were related to suicidality in the present study, they leaned towards warning signs more than risk factors. They appeared to be proximal and precipitating factors, which were elevating the suicide crisis to an active one, more than an enduring, distal, and static factor. All the suicide attempts in the present study arose from life’s crises experienced without early intervention. This becomes an important issue because, people may still believe that suicide happens without warning signs. It becomes glaring in the face of the repeated statements from most of the family members that they could not understand why their relative wanted to die. This finding is consistent with Frey, Hans and Cerel [7] where reactions from family and friends indicated that suicide attempt survivor was a burden. This burden according to Joiner [1] can lead to suicidal behaviour.

Research shows that most suicides give many clues and warnings signs about their suicidal intentions [15]. Warning signs, therefore, become very important for making quick decisions about the current state of the individual and the help the person may need [25, 26]. In light of mental health workforce shortages and mental health system inadequacies in Ghana, educating lay public is an invaluable asset in improving awareness and increasing opportunities for early detection, support and intervention. In five cases in the present study, the families were utterly ignorant of what led to the suicide attempt. It generally shows that they were uninformed about the warning signs for suicide attempts. We can speculate that the widespread negative attitudes toward suicide in Ghana including condemnation, insults, social taunting, physical abuse of the attempt survivor and others may act as barriers to communication of intent and early detection of suicide warning signs [12]. As reported, stigma is a significant barrier to early detection of suicide warning signs [16]. Consistent with such reflections and the present study, Leenaars’ [15] profound statement about the inability of people to detect suicide warning signs is instructive:

*Suicide is not just determined by the present; it has a history. Regrettably, the clues to suicide are usually not seen, heard, or even responded to before the act. People—spouse, parents, teacher, siblings, elders, and so on—have not seen the history. (p. 222)*

Much as we cannot explicitly assert that families who confirmed the reasons of the attempt survivor may have detected warning signs early enough, we can implicitly assume that they might have communicated with the attempt survivors and been accordingly informed about their distress. Nonetheless, they may not have intervened and subsequently could not prevent the attempt. This might be accounted for by the fact that knowledge of warning signs does not automatically translate into competency in intervening due to discomfort, uncertainty, hopelessness about ability to effectively intervene as well as the barrier posed by social stigma [16]. Rudd has consequently, advised that “it is important for any effort to train individuals to identify warning signs be coupled with targeted training on how to intervene and how to access emergency services” [25].

The similar and different reports of the reasons for suicide also point toward suicide communication as perceived in general. It has been indicated that suicide attempts have many meanings and attempters may not necessarily want to die but rather communicate the intense pain to those around [15]. Parallel to the proposition of others [27] we view the suicidal attempts in the present study as a part of a broader concept such as a reaction to crises than single dependent variable. Thus, the reasons for the attempt points towards goal-directed suicides. This was seen in the fact that almost all the attempt survivors were engaging in suicide to solve one problem or another ranging from domestic abuse, sense of loss, dyadic tensions among others. Such observation is different from the popular reading of suicide attempts as sequelae to mental illness, and hence a pathology [28, 29]. Importantly, whereas family members certainly have their own perspectives on the reasons for a suicide attempt, the suicide attempt survivor is better positioned to understand their own “lived experience” and family members and healthcare providers should strive to understand the survivor’s perspective.

The findings in the present study have implications for suicide prevention in Ghana. Consistent with Shneidman [30] and Leenaars [15], an important approach to suicide prevention is public education about clues and warning signs and information about what to do. The general public including families with incidents of suicide attempt needs to be engaged in seeking to improve their understanding about suicide through education. There is a dearth of this approach in the country, but studies continue to highlight the need for such an approach [14]. A critical dimension of such public education is to emphasise and promote the need for communication between close knitted relations especially when someone in such a network is in suicidal distress. This can be an important early suicide detection intervention goal in Ghana.

The second significant implication of the findings of the present study is for clinical practice. In working with attempt survivors, it might be helpful to involve close family members in order to improve the communication and understanding of the suicidal process as well as supportive responses from the relatives. In an extensive review of interventions for suicidal youth, family or close relations’ intervention is found to be very useful [31]. Such an approach may help address specific dysfunctional issues in close relations and strengthen the supportive network during a suicidal crisis. Mental health workers in Ghana, providing help to suicide attempt survivors can inform their intervention schemes with this finding.

### Limitations

A limitation of the study is the use of only suicide attempt survivors and families without involving other parties like friends and therapists of the suicide attempt survivor. The involvement of such groups may further our understanding of the reasons for the attempt. This is because in a proscriptive culture like Ghana, people may prefer to confide in their friends and health providers rather than family members because of fear of negative judgment. Thus, probably we could have discovered other dimensions of the reasons for the suicidal action than we have uncovered in the present study. Another limitation is interviewing families referred to us by the attempt survivor. This may be biased and might have affected responses given by the families. However, the findings in the present study may be initially useful signposts for more robust future studies.

## Conclusions

The study has demonstrated the important role attempt survivor family members could play in the early detection and intervention of suicidal crisis in Ghana. One important indication is that attempt survivor family members need suicide literacy in order to efficiently communicate with their wards. It will be most useful to engage such families in any therapeutic interventions and the implication is that context is extremely important in any suicide intervention programs.

## Additional file

Additional file 1: Interview guide. (DOCX 15 kb)

## Abbreviation

WHO: World Health Organization
