# Supplementary material for: Comparing the reasons for suicide from attempt survivors and their families in Ghana
Source: BMC Public Health. 2019 Apr 16;19:412. doi: 10.1186/s12889-019-6743-z (PMC6469126; doi:10.1186/s12889-019-6743-z)
Supplement: Supplementary file 1 — Interview guide. (DOCX 15 kb) [file 12889_2019_6743_MOESM1_ESM.docx]

**INTERVIEW SCHEDULE- FAMILY**

1. How prevalent do you think suicide is in Ghana today?
2. Do you think it will change in the future? That people will stop committing suicide?
3. How do you think topic of suicide is viewed in Ghana today? Do you think people stigmatize suicidal people?
4. In general do people talk about suicide?
5. Do you think suicide is talked about in the media?
6. And in the community, the neighborhood where you live.
7. Have you had a suicidal attempt/death in your family?
8. Do you know why it happened? Why do you think your relative attempted suicide?
9. How does it feel to hear a close relation has attempted suicide?
10. So did you feel said, guilty, depressed?
11. Describe how people reacted to the attempt?
12. How did the hospital staff treat the case and how people treated the case?
13. Can you tell how the family folks reacted to it?
14. Did you tell anyone at church or get support from church?
15. Why?
16. Tell me how you managed the feelings?
17. Were there people helping you to cope?
18. Can suicidal behavior be justified? That is can we ever say there is one particular reason that someone engages in suicide is understandable.
19. What is your view about the fact that the person thinks he or she has the right to take his life as and when he or she feels ok to do so?
20. Tell me the reasons or circumstances under which you think suicidal behavior can be justified or acceptable?
21. Are you aware that attempted suicide is a crime?
22. Do you think it should still be considered a crime (as it is at present according to Ghanaian law. Please provide reasons)?
23. According to the present law, what would you have wished was done to your relative who attempted suicide? (Explain your response).

16. Do you think suicide can be prevented? Explain

1. Do you think suicide should be prevented? Explain
2. If so, how can it be done and who could contribute?
3. Closure and debriefing
4. Do you have anything else to tell me about this topic?
5. Do you have any questions for me?
6. How did you feel about being interviewed on this topic?
7. Is there anything you wish had been done differently
8. Did the suicide attempt affect your work?

**INTERVIEW SCHEDULE- SUICIDE ATTEMPTER**

1. How has your life been?

2. How would you describe your situation in your family?

3. Do you feel important/appreciated in your family? How?

4. How do you feel about your community?

5. How is your relationship with your colleagues/classmates?

6. Do you feel appreciated at school/work?

7. Did you have problems at work/in school?

8. In your own words, please tell me what led to you attempt suicide? Why did you engage in the suicidal behaviour?

9. Can you please describe your feelings before the suicide attempt/self-harm?

10. Did you feel any pain?

11. How did you feel after you harmed yourself/the suicide attempt?

12. What were you hoping for and how did you cope after?

13. Have you tried to take your own life or harmed yourself before?

14. Has anyone in your family ever attempted or completed suicide?

15. Did this influence your own suicide attempt in any way?

16. Do you know of anyone in your community who has attempted or completed suicide?

17. Did this influence your own suicide attempt in any way?

18. Is there any history of alcohol/drug abuse in your family?

19. Do you normally use alcohol/drugs?

20. Was alcohol/drugs involved in your suicide attempt?

21. Did you ever experience any abuse?

22. Would you describe yourself as religious?

23. How do you feel about your church/mosque/religious institution?

24. Is your relationship to God important to you?

25. How does your relationship to God influence your life?

26. Did your suicide attempt have anything to do with your relationship to God or your church?

27. Does your suicide attempt have any influence on your relationship to God?

28. How did your family and close friends treat you when they found out that you had harmed yourself/attempted to kill yourself?

29. Did they treat you in the way you expected or wanted them to?

30. When you were admitted to the hospital after your self-harm/suicide attempt, how did the hospital staff treat you?

31. Did they treat you in the way you expected or wanted them to?

32. How do you think about killing/harming yourself now? Would you consider doing it again?

1. If yes: What would you need in order not to think about killing yourself?
2. If no: What has made you change your mind?

33. At the time you harmed yourself/attempted suicide, did you consider any consequences this act might have?

34. Do you think that the law criminalizing attempted suicide is fair?

35. Is there anything that you deem important for understanding your situation and your suicide attempt that I have not asked about and that you want to tell me?
